# Mechanisms of Modulation of Ferroptosis and Its Role in Central Nervous System Diseases

**DOI:** 10.3389/fphar.2021.657033

**Published:** 2021-06-04

**Authors:** Qingyun Tan, Yuying Fang, Qiong Gu

**Affiliations:** Research Center for Drug Discovery, School of Pharmaceutical Sciences, Sun Yat-sen University, Guangzhou, China

**Keywords:** ferroptosis, lipid metabolism, iron metabolism, glutathione peroxidase 4, coenzyme Q_10_, central nervous system diseases

## Abstract

Ferroptosis is a new form of programmed cell death characterized by intracellular iron-dependent accumulation of lipid peroxide and primarily associated with iron metabolism, glutathione-dependent pathway, and coenzyme Q_10_-dependent pathway. Recent studies demonstrate that ferroptosis is associated with central nervous system (CNS) diseases, such as stroke, Parkinson’s disease, Alzheimer’s disease, and Huntington’s disease. This review summarizes the key regulatory mechanisms of ferroptosis and its role in CNS diseases. These updates may provide novel perspective for the development of therapeutical agents against CNS diseases.

## Introduction

From organisms to cells, death is the common destiny of life. Conventional cell death removes damaged or harmful cells from organisms. Therefore, cell death is essential for the homeostasis of life. When cell death is over-activated, the body can suffer from many pathological conditions, such as nervous system diseases. Thus, understanding the process of cell death helps to intervene in cell death or survival and develop therapeutical solutions to treat associated diseases.

The major forms of cell deaths are divided into apoptosis, autophagy, and necrosis. Recently, morphological and biochemical criteria have been generated to articulate cell death and mechanisms. New cell death forms are discovered from time to time, such as pyroptosis (Fink and Cookson, 2005) and ferroptosis (Dixon et al., 2012).

Before the term “ferroptosis” was formally proposed, many scientists had observed this form of cell death (Wolpaw et al., 2011; Yagoda et al., 2007). Without a systematic concept and detailed molecular knowledge, ferroptosis was attributed to the other existing forms of cell death and was considered to have no biological significance. In 2012, Dixon et al. (2012) found that erastin, a small molecule inducer, could induce *RAS*-mutated tumor cell death by overwhelming lipid peroxidation that produced lipid reactive oxygen species (ROS, a class of highly reactive chemical molecules formed from the electron receptivity of O_2_). This cell death form depends on iron rather than other metals, and can be suppressed by iron chelator deferoxamine (DFO). Therefore, such cell death was termed as “ferroptosis” by Dixon and co-workers. Since then, ferroptosis has drawn a great attention. It was found vital to many pathophysiological conditions, such as nervous system diseases (Derry et al., 2020), ischemia/reperfusion injury (Guan et al., 2019), tumor (Shin et al., 2020) and acute kidney injury (Ma et al., 2020).

Based on the morphological, biochemical, and genetic characteristics, ferroptosis is distinct from other forms of cell death. Ferroptotic cells have smaller mitochondria with reduced crest, condensed membrane density and ruptured outer membranes (Dixon et al., 2012). The cells do not show rupture and blebbing on the plasma membranes, the features of apoptotic cells (Xie et al., 2016). Using transmission electron microscopy, the ferroptotic cells can be distinguished from other forms of cell death. The main biochemical characteristic of ferroptosis is iron-dependent over-oxidation of polyunsaturated fatty acids (PUFAs)-containing phospholipids (PLs) on cell membranes (Dixon et al., 2012). Apoptosis has long benefited from the detection of the cleaved caspase-3. However, it is unclear what are the biomarkers (either transcriptional up-regulation or, post-translational modification of specific cell death effectors or, pore-forming proteins) required for the final execution of ferroptosis (Hirschhorn and Stockwell, 2019). Genetically, Dixon and his colleagues (Dixon et al., 2012) found that the expression of many genes changed in erastin-induced ferroptosis, including ribosomal protein L8 (*RPL8*), iron response element binding protein 2 (*IREB2*), ATP synthase F0 complex subunit C3 (*ATP5G3*), citrate synthase (*CS*), tetratricopeptide repeat domain 35 (*TTC35*) and acyl-CoA synthetase family member 2 (*ACSF2*). Meanwhile, many genes involved in the regulation of apoptosis and other non-apoptotic cell death were not altered. Subsequently, more genes are being found to be associated with ferroptosis, such as heat shock protein β-1 (*HSPB1*) (Sun et al., 2015) and *p53* (Jiang et al., 2015). Importantly, the expression of prostaglandin-endoperoxide synthase 2 *(PTGS2)* was found to be significantly upregulated in ferroptosis without changing ferroptosis process (Yang et al., 2014). Thus, this gene expression is regarded as a biomarker of ferroptosis and utilized to distinguish ferroptosis from other forms of cell death.

In this review, we summarize the mechanisms of modulation of ferroptosis and its role in central nervous system (CNS) diseases, and propose the possible strategies for finding new ferroptosis regulators.

## The key regulatory mechanisms of ferroptosis

**FIGURE 1:**
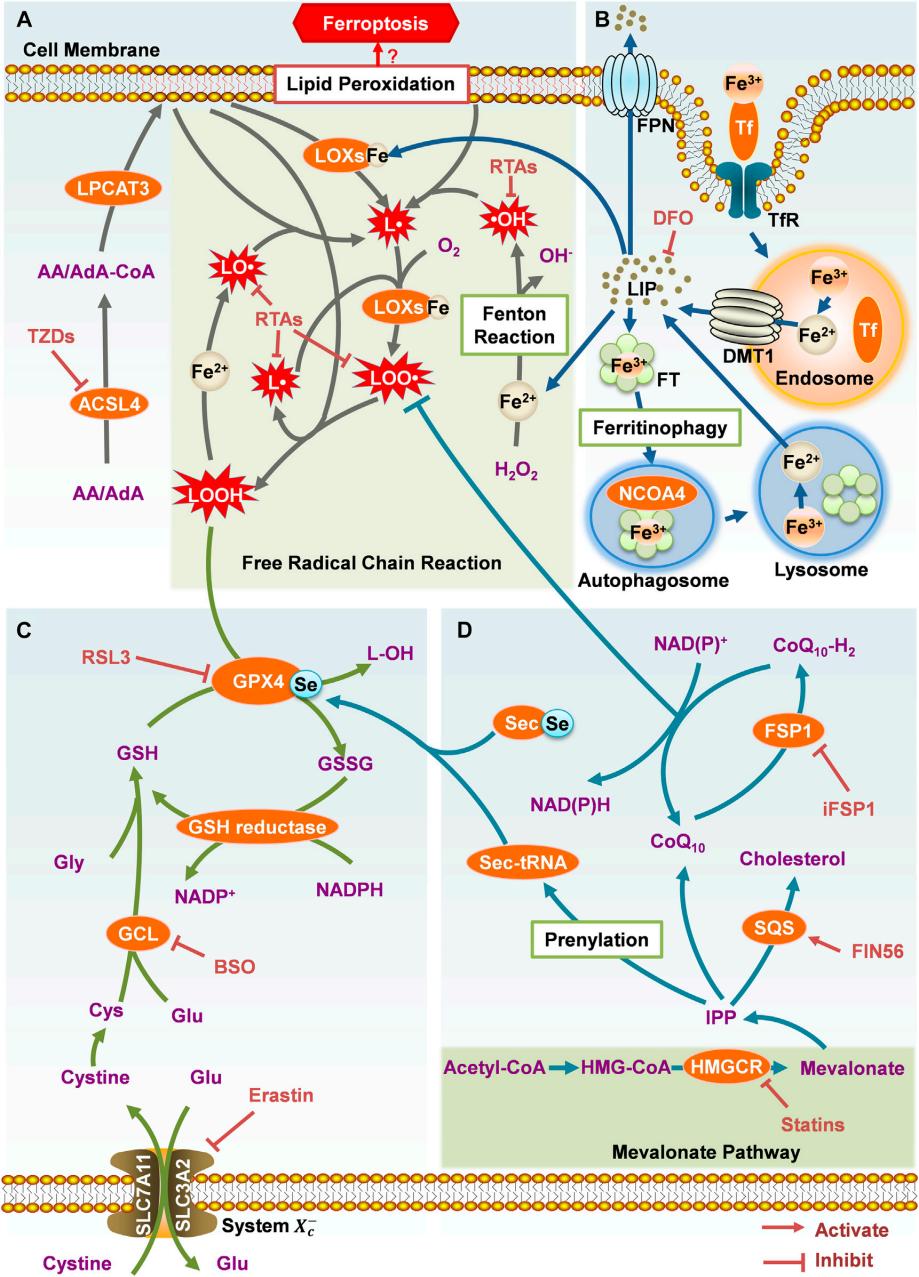
The key regulatory mechanisms of ferroptosis. **(A)** Lipid metabolism. **(B)** Iron metabolism. **(C)** Glutathione-dependent pathway. **(D)** CoQ_10_-dependent pathway.

The essence of ferroptosis is intracellular excessive lipid peroxidation and the metabolic disorders of its product lipid hydroperoxides (LOOHs) (Figure 1A). With iron as the catalyst, a large number of LOOHs are produced to destroy the intracellular redox balance and attack biological macromolecules, finally triggering cell death. These LOOHs are crucial factors to execute ferroptosis, and inhibiting their formation can suppress cell death. It is noteworthy that the lipid peroxidation and metabolic disorders of intracellular LOOHs are mainly related to iron metabolism (Figure 1B), glutathione (GSH)-dependent pathway (Figure 1C), and coenzyme Q_10_ (CoQ_10_)-dependent pathway (Figure 1D). We will elaborate on these mechanisms below.

However, the mechanism that ultimately leads to ferroptotic cell death is still unclear, so further research is needed. There are two prevalent hypotheses: 1) Lipid peroxidation and ROS over-production destroy cell membrane integrity through damaging and perforating the cell membranes (Agmon et al., 2018); 2) LOOHs are decomposed into toxic aldehydes, such as 4-hydroxy-2-nonenal (4-HNE) and malondialdehyde (MDA), which crosslink and dysfunction the proteins required for cell viability, resulting in cell death (Zhong and Yin, 2015; Angeli et al., 2017).

### Lipid Metabolism

Compared with saturated fatty acids (SFAs) and monounsaturated fatty acids (MUFAs), PUFAs are easier to be oxidized. This is because the double bond near the *bis*-allyl methylene group in PUFAs can weaken the hydrogen bonding energy of the methylene group, resulting in its sensitivity to dehydrogenation and subsequent oxygenation (Else, 2017). A recent study (Kagan et al., 2017) demonstrated that, when containing the two types of PUFAs arachidonic acid (AA) and adrenic acid (AdA), the PLs especially phosphatidylethanolamines (PEs) on the cell membranes, are more susceptible to be oxidized. The over-oxidation can lead to ferroptosis eventually.

**FIGURE 2:**
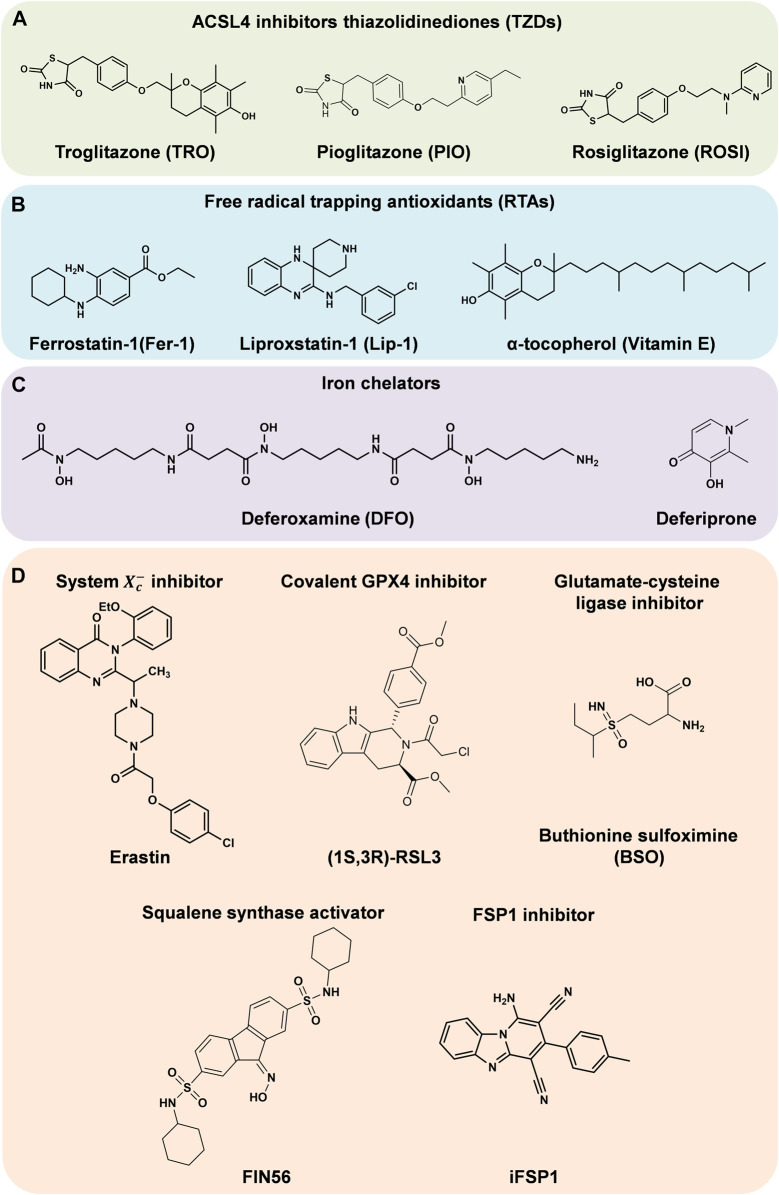
Regulators of ferroptosis. **(A–C)** Inhibitors of ferroptosis. **(D)** Inducers of ferroptosis.

Before the initiation of lipid peroxidation, intracellular free AA and AdA need to be inserted into the PLs on the cell membranes. First, free AA and AdA are activated by acyl-CoA synthetase long-chain family member 4 (ACSL4) to form AA-CoA and AdA-CoA. Subsequently, they are inserted to PLs by esterification reaction under the catalysis of lysophosphatidylcholine acyltransferase 3 (LPCAT3) (Magtanong and Dixon, 2018). Consequently, the easily oxidized membrane PLs are synthesized, which are more likely to cause the lethal lipid peroxidation and ferroptosis. Therefore, the activities of these two enzymes are important to the cellular sensitivity to ferroptosis. The ACSL4 inhibitors thiazolidinediones (TZDs) include troglitazone (TRO), pioglitazone (PIO) and rosiglitazone (ROSI) (Figure 2A). They were reported to suppress ferroptosis in mouse embryonic fibroblasts (Doll et al., 2017). Knockdown of *Lpcat3* could also make mouse lung epithelial cells and embryonic cells more resistant to ferroptosis (Kagan et al., 2017). When ACSL4 or LPCAT3 are inhibited, the available substrates of lipid peroxidation are reduced and the lipid peroxidation is suppressed. ACSL4 and LPCAT3 are promising targets against ferroptosis or other peroxidation related diseases.

Lipoxygenases (LOXs) are iron-containing enzymes for cell membrane PLs oxidation, which are non-heme dioxygenases that catalyze the double oxygenation reaction of free and esterified PUFAs (Brash, 1999). Different subtypes of LOXs catalyze the dioxygenation of PUFAs at different positions. Baicalein, a naturel bioactive compound, was reported to inhibit ferroptosis by suppressing 12/15-LOX (Li et al., 2019). The first step of LOXs catalysis is to abstract an unstable hydrogen from the *bis*-allyl position at a PUFA to form a pentadienyl radical (L·) (Kuhn et al., 2015). This step can also be accomplished by an auto-oxidation reaction independent of LOXs. Highly reactive substances such as hydroxyl radicals (·OH), alkoxy groups (LO·) and hydroperoxyl radicals (HO_2_·) can take a hydrogen atom from the *bis*-allyl position of a PUFA (Angeli et al., 2017). Subsequently, molecular oxygen is added to the carbon-centered radicals to yield a peroxy radical (LOO·). The LOO· can abstract a hydrogen atom from an adjacent PL to generate a LOOH and a new L·. As a result, this free radical chain reaction continues to propagate and generates more LOOHs (Yin et al., 2011). Additionally, LOOHs can be oxidized by Fe^2+^: the O-O bonds are broken and yield LO·. The LO· participates in the free radical chain reaction, destroys the adjacent PUFAs directly, and causes cell membrane damage and ferroptosis (Gaschler and Stockwell, 2017).

With Fe^2+^, Fenton reaction converts hydrogen peroxide (H_2_O_2_) to ·OH radicals, which propagate free radical chain reactions (Ayala et al., 2014). Radical trapping antioxidants (RTAs) provide electrons to neutralize free radicals (Hassannia et al., 2019). This suppresses the propagation of lipid peroxidation and act as ferroptosis inhibitors. Such ferroptosis inhibitors include ferrostatin-1 (Fer-1) (Dixon et al., 2012), liproxstatin-1 (Lip-1) (Angeli et al., 2014) and α-tocopherol (vitamin E) (Kajarabille and Latunde-Dada, 2019) (Figure 2B).

### Iron Metabolism

The demand for iron is a defining characteristic of ferroptosis. Since Fe^2+^ catalyzes Fenton reaction, and also is an essential component of ROS-producing enzymes such as LOXs and NADPH oxidase, iron affects lipid peroxidation and cellular sensitivity to ferroptosis. Increasing the content of free Fe^2+^ in cells advances their sensitivity to ferroptosis. Conversely, iron chelators (Figure 2C) and other substances that can reduce the concentration of intracellular iron are able to inhibit ferroptosis. Under physiological conditions, cellular iron homeostasis is regulated through iron uptake, storage and export.

Transferrin (Tf)-mediated iron transport is the most important way of cellular iron uptake. It can transport Fe^3+^ from the place where Fe^3+^ is absorbed and stored to the iron-requiring site of the body. The Tf carrying Fe^3+^ is recognized by the transferrin receptor (TfR) on the cell membranes and endocytosed into the cells. Fe^3+^ is released from the Tf in the acidic environment of endosomes and reduced to Fe^2+^ by ferrous reductase. Afterwards, Fe^2+^ is transported to the cytoplasm through divalent metal transporter 1 (DMT1) on the endosomal membranes (Ji and Kosman, 2015). The free Fe^2+^ forms a labile iron pool (LIP) and plays its physiological or pathological roles. Inhibiting the iron uptake could reduce the level of LIP and suppress ferroptosis. For example, either immuno-depletion of Tf in serum or RNAi of *TfR* could significantly inhibit ferroptosis in mouse embryonic fibroblasts (Gao et al., 2015).

The excess Fe^2+^ in the cells would be stored in ferritin (FT) to maintain the content of iron under normal physiological conditions. FT is a hollow globular protein shell composed of two types of subunits: ferritin heavy chain 1 (FTH1) and ferritin light chain (FTL) (Harrison and Arosio, 1996). Each FT can store about 4,500 Fe^3+^ in the form of Fe_2_O_3_·nH_2_O (Islam et al., 1989). Oncogene-*RAS*-harboring cancer cells are more sensitive to ferroptosis, partly because *RAS* can down-regulate the expression of FTH1 and FTL, increasing intracellular LIP (Yang and Stockwell, 2008). Recent studies indicated that nuclear receptor coactivator 4 (NCOA4)-mediated ferritinophagy played a crucial role in the regulation of iron levels. When available iron in cells is scarce, NCOA4 would recognize and bind to FTH1, and then recruit FT to autophagosomes. With the formation of autolysosomes, FT complexes enter lysosomes and are degraded, subsequently Fe^3+^ stored in these complexes would be released and supply the LIP. This process is also necessary for the execution of ferroptosis. Silencing the expression of NCOA4 by RNAi knockdown significantly inhibited ferritinophagy, thereby suppressing ferroptosis in mouse embryonic fibroblasts (Gao et al., 2016).

In addition, excess intracellular Fe^2+^ can also be exported through ferroportin (FPN) on the cell membranes, which is the only known vertebrate iron efflux pump (Bogdan et al., 2016). By regulating FPN, the content of intracellular Fe^2+^ can be changed, and ferroptosis can be mediated. It was reported that knockdown of *Fpn* in neuroblastoma cells could increase the accumulation of iron-dependent lipid ROS, and thereby accelerate erastin-induced ferroptosis (Geng et al., 2018). Overexpression of *FPN* abolished the erastin-induced ferroptosis in ectopic endometrial stromal cells (Li et al., 2021). In the brains of Alzheimer's mouse model, genetic deletion of *Fpn* increased ferroptosis and then induced memory impairment, while restoring *Fpn* ameliorated ferroptosis and memory impairment (Bao et al., 2021).

### GSH-dependent Pathway

Glutathione peroxidase 4 (GPX4) is a selenium (Se)-containing enzyme, which plays a central role in the reduction of lipid ROS production. With consumption of two GSH molecules, GPX4 could reduce toxic LOOHs to non-toxic lipid alcohols (L-OHs). However, when GPX4 is deficient or inactive, LOOHs will accumulate to a high level, leading to catastrophic membrane damage. It is currently believed that inhibiting GPX4 by direct or indirect ways is the key to induce ferroptosis.

The ways to inhibit GPX4 directly mainly include covalently binding GPX4 and suppressing its expression. The compound Ras-selective lethal 3 (RSL3, Figure 2D), which can covalently bind to the selenocysteine (Sec) at the active site of GPX4 and inhibit its activity, is a highly effective ferroptosis inducer (Dixon et al., 2012). Knockout of *Gpx4* can promote ferroptosis in mouse embryonic fibroblasts, while overexpression of *Gpx4* made cells more resistant to RSL3-induced ferroptosis (Yang et al., 2014).

Inhibiting GPX4 indirectly mainly involves inhibition of its cofactor GSH production. GSH is synthesized from three amino acids: glutamate (Glu), cysteine (Cys) and glycine (Gly). Among them, the amount of Cys is usually the least in cells, so it is considered to be the key factor limiting the *de novo* synthesis of GSH. Cys exists in its oxidized form cystine outside the cells. Through cystine/Glu antiporter (system $X_{c}^{-}$) on the cell membranes, an extracellular cystine is transported into cells, and meanwhile an intracellular Glu is exported. The system $X_{c}^{-}$ is a disulfide-link heterodimer consisting of SLC7A11 (xCT) and regulatory subunit SLC3A2 (4F2hc and CD98hc) (Sato et al., 1999). This transport process does not depend on ATP but is driven by the concentration difference of Glu or cystine on both sides of the membranes. Although Cys can be generated via the transsulfuration pathway in some cell types, in many other cell types, at least *in vitro*, the import of cystine via system $X_{c}^{-}$ is significant for maintaining the levels of Cys and GSH, and preventing ferroptosis (Magtanong and Dixon, 2018). When this transport is impaired, GSH will be depleted, making GPX4 unable to reduce LOOHs. For example, erastin (Figure 2D), a potent inducer of ferroptosis, is a specific inhibitor of the system $X_{c}^{-}$ (Dixon et al., 2012); the deletion of a system $X_{c}^{-}$ subunit *Slc7a11* in mice induces ferroptosis and inhibits the growth of pancreatic ductal adenocarcinoma (Badgley et al., 2020); high concentration of extracellular Glu inhibits the import of cystine and promotes ferroptosis, which is termed “oxidative glutamate toxicity” in neurons or neuronal-like cells (Magtanong and Dixon, 2018); cystine deprivation suppresses the growth of head and neck cancer by promoting ferroptosis (Shin et al., 2020). Additionally, inhibiting the synthesis of GSH can also promote ferroptosis. The compound buthionine sulfoximine (BSO, Figure 2D) can induce ferroptosis in retinal pigment epithelium by inhibiting glutamate-cysteine ligase (GCL), a rate-limiting enzyme in *de novo* GSH synthesis (Sun et al., 2018).

### CoQ_10_-dependent Pathway

However, the responses of GPX4 inhibitors in different cell lines are not consistent (Zou et al., 2019), indicating that there may be pathways independent of GPX4 to regulate ferroptosis.

Researchers have found that the mevalonate pathway could also affect ferroptosis. Isopentenyl pyrophosphate (IPP) is a direct metabolite of mevalonate, which can be used for Sec-tRNA prenylation, CoQ_10_ synthesis and cholesterol biosynthesis (Moosmann and Behl, 2004). On the one hand, only the prenylated Sec-tRNA can carry Sec to GPX4, complete the synthesis of GPX4 (Warner et al., 2000), and then inhibit ferroptosis (Yang and Stockwell, 2016); on the other hand, the reduced form of CoQ_10_ (CoQ_10_-H_2_) is a potent lipophilic antioxidant, which can capture LOO· to prevent the spread of free radical chain reaction and inhibit the production of LOOHs, and meanwhile the CoQ_10_-H_2_ is oxidized (Bentinger et al., 2007). Therefore, inhibiting Sec-tRNA prenylation and CoQ_10_ synthesis will disrupt GPX4 synthesis and CoQ_10_-H_2_ antioxidant activity respectively, and eventually induce ferroptosis. For instance, FIN56 (Figure 2D) can activate squalene synthase (SQS), a key enzyme in cholesterol biosynthesis^39^, and then suppress Sec-tRNA prenylation and CoQ_10_ synthesis, finally leading to ferroptosis in human fibrosarcoma HT1080 cells (Hassannia et al., 2019; Shimada et al., 2016b). 3-Hydroxy-3-methyl glutaryl-coenzyme A (HMG-CoA) reductase (HMGCR) is an important enzyme in the mevalonate pathway. Statins, as a type of inhibitors of HMGCR, can promote the lethality of FIN56 (Shimada et al., 2016b).

Unless maintaining in the reduced state, the oxidized CoQ_10_ is unable to inhibit the spread of LOOHs. In 2019, Doll et al. (2019) and Bersuker et al. (2019) conducted an overexpression screen and a synthetic lethal CRISPR-Cas9 knockout screen, respectively. Both groups revealed that ferroptosis suppressor protein 1 (FSP1) could suppress ferroptosis when knockout or inhibit GPX4. FSP1 is essentially a CoQ_10_ oxidoreductase, which utilizes NAD(P)H to catalyze the reduction of CoQ_10_, maintaining the availability of CoQ_10_-H_2_. Bersuker et al. (Bersuker et al., 2019) found that the expression level of FSP1 was positively correlated with ferroptosis resistance in hundreds of cancer cell lines. Besides, in tumor xenograft mice model, the growth of *GPX4* ^KO^ *FSP1* ^KO^ tumors was suppressed, while *GPX4* ^KO^ tumors grew normally. Through screening nearly 10,000 drug-like compounds, Doll et al. (Doll et al., 2019) identified the first effective FSP1 inhibitor iFSP1 (Figure 2D). HT1080 and mouse Pfa1 treated with iFSP1 were much more sensitive to ferroptosis. In conclusion, by regulating the redox of CoQ_10_, FSP1 acts as an essential component of the non-mitochondrial CoQ_10_ antioxidant system, as well as an enzyme catalytic system that is able to complement the loss of GPX4 in cells.

### Other Factors That Regulate Ferroptosis

**FIGURE 3:**
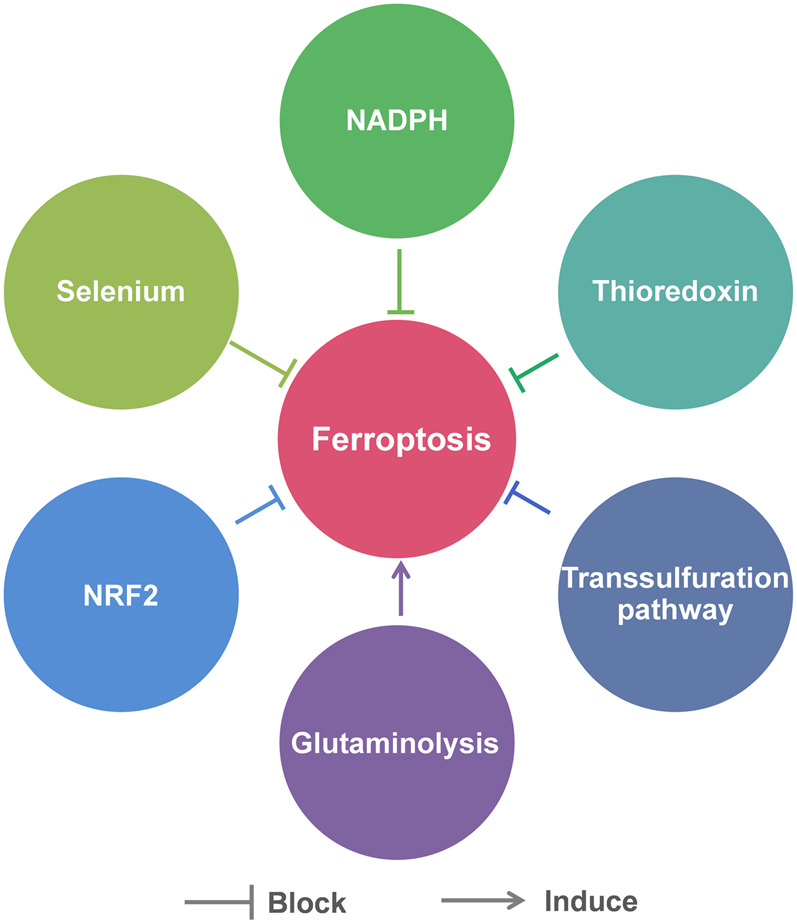
Other factors that regulate ferroptosis.

Besides the above pathways, there are many other factors that are involved in the regulation of ferroptosis, including Se, NADPH, thioredoxin, transsulfuration pathway, glutaminolysis and nuclear factor erythroid 2-related factor 2 (NRF2) (Figure 3).

Se is currently recognized as an essential micronutrient beneficial to health. Its beneficial effects are mainly due to its incorporation into selenoprotein in the form of Sec (Friedmann Angeli and Conrad, 2018). Sec is similar to cysteine, in which sulfur is replaced by Se. As mentioned previously, Sec is an important component of GPX4. Therefore, Se influences cellular sensitivity to ferroptosis to some extent. It was reported that Se deprivation significantly increases oxidative stress in cells and their susceptibility to ferroptosis (Cardoso et al., 2017). Moreover, Ingold et al. (2018) generated mice with targeted mutation of the active site Sec to Cys of GPX4. They found the *Gpx4* ^*cys/cys*^ mouse embryonic fibroblasts were extremely sensitive to peroxide-induced ferroptosis. In addition to directly participating in the synthesis of selenoproteins, Se may increase the resistance of cells to ferroptosis in indirect ways. A study showed that Se supplement could stimulate transcriptional adaptive program of cells to synthesize more antioxidant selenoproteins, including GPX4 and thioredoxin reductase 1 (TXNRD1), to block ferroptosis (Alim et al., 2019).

NADPH can also modulate ferroptosis by indirectly affecting the activity of GPX4, due to that it is a vital reductant in the process of GSH production. The abundance of basal NADP(H) in cells is positively related to the resistance to ferroptosis. It was reported that knockdown of NAD^+^ kinase (NADK), an enzyme that uses NAD^+^ to synthesize NADP(H), was able to decrease NADP(H) levels in HT1080 cells and make them more susceptible to ferroptosis inducers (Shimada et al., 2016a). In addition, NADPH oxidase (NOX) family, which is able to decrease the available level of intracellular NADPH, was found to be upregulated in several *RAS* mutant tumors (Kamata, 2009). Diphenylene iodonium (DPI), a NOX inhibitor, was found to prevent erastin-induced ferroptosis in *KRAS* mutant Calu-1 non-small cell lung cancer cells (Dixon et al., 2012).

Thioredoxin, a member of cellular antioxidant family (Nordberg and Arner, 2001), plays an important role in suppressing ferroptosis by maintaining and regulating the redox homeostasis. In a recent study, Llabani et al. (2019) performed structural modification of the natural product pleuromutilin and synthesized a series of diverse compounds. Through phenotypic screen and biological evaluation, they discovered that the small molecule ferroptocide could induce lipid peroxidation and ferroptosis in some tumor cell lines. Subsequent studies identified ferroptocide is a covalent inhibitor of thioredoxin. This group also demonstrated that knockdown of thioredoxin led to massive generation of general and lipid ROS in HCT 116 colon cancer cells.

As mentioned above, in some cell types, cysteine can be generated through the transsulfuration pathway rather than system $X_{c}^{-}$. When the intracellular cysteine is insufficient, methionine will act as a sulfur donor and undergo a series of reactions to produce cysteine, which can be used for the synthesis of GSH. Therefore, these cells are not sensitive to ferroptosis induced by inhibitors of system $X_{c}^{-}$. For example, Hayano et al. (2016) found that activation of transsulfuration pathway in HT1080 cells could increase their resistance to erastin-induced ferroptosis. Inversely, Wang et al. (2018) designed and synthesized a compound named CH004 as an inhibitor of cystathionine β-synthase, which catalyzes the first enzymatic reaction in the transsulfuration pathway. They found that compound CH004 triggered ferroptosis in hepatocellular carcinoma HepG2 cells and significantly suppressed tumor growth in a xenograft mice model bearing H22 mouse liver tumor cells.

Glutaminolysis is the metabolism of intracellular glutamine, through which cells use glutamine as a carbon source for the mitochondrial tricarboxylic acid (TCA) cycle as well as a nitrogen source for the synthesis of certain necessary substances. Gao et al. (2015) found that glutaminolysis was necessary for ferroptosis induced by cystine deprivation: either RNAi knockdown of glutamine influx receptor SLC1A5 or glutaminolysis inhibitor Compound 968 could inhibit cystine deprivation-induced ferroptosis. Mechanistically, the TCA cycle and electron transport chain in mitochondria drive this type of ferroptosis. Inhibition of glutaminolysis could suppress the TCA cycle, the hyperpolarization of mitochondrial membrane potential and the accumulation of lipid ROS, eventually inhibit ferroptosis (Gao et al., 2019). This finding also confirmed the vital role of mitochondria in ferroptosis, which had been long-term controversial.

NRF2, a member of basic leucine zipper transcription factors, is a key regulator of cellular antioxidant response, because its target genes include some antioxidant proteins/enzymes genes. Sun et al. (2016) revealed the p62-Kelch-like ECH-associated protein (Keap1)-NRF2 antioxidative signaling pathway involved in the ferroptosis resistance in hepatocellular carcinoma cells. They found that p62-mediated degradation of Keap1 could promote NRF2 activation. Thus, the genes NAD(P)H quinone oxidoreductase-1 (*NQO1*), heme oxygenase-1 (*HO1*) and *FTH1* regulated by NRF2 protected the cells from ferroptosis by modifying lipid peroxidation and iron metabolism. In addition, it was reported that the cells with higher expression levels of auxin response factor (ARF) were more susceptive to ferroptosis, as ARF could inhibit the ability of NRF2 to activate its target genes, including SLC7A11 (Chen et al., 2017). Recently, a NRF2-Focadhesin (FOCAD)-focal adhesion kinase (FAK) signaling pathway was proposed. FOCAD-FAK signaling was able to make non-small-cell lung carcinoma cells more sensitive to cysteine deprivation-induced ferroptosis, while NRF2 could negatively regulate the pathway (Liu et al., 2020). These findings underlined the role of NRF2 in ferroptosis.

## Ferroptosis and Central Nervous System Diseases

With the in-depth study of ferroptosis, its therapeutic potentials have also received widespread attention. It has been widely reported that ferroptosis inducers can potently kill tumor cells and inhibit tumor growth in mouse xenograft tumor models, which indicates that ferroptosis inducers are enormously potential in human cancer treatments (Hassannia et al., 2019; Mou et al., 2019). For instance, Hassannia et al. (2018) identified withaferin A as a natural ferroptosis inducer in neuroblastoma, which could inhibit the *in vivo* growth and recurrence rate of neuroblastoma xenografts. However, ferroptosis was also found to cause neuronal death in rat organotypic hippocampal slice culture (OHSC) models, showing the harmful pathological effect of ferroptosis (Dixon et al., 2012). Moreover, the cells in CNS are more susceptible to ROS toxicity owing to their inherent more membranous fatty acids and less antioxidant enzymes, as well as higher oxidative metabolism (Olmez and Ozyurt, 2012). Increasing evidence (Weiland et al., 2019) indicates that ferroptosis may be a driver in some CNS diseases caused by the dysfunction and cell death in CNS, such as stroke, Parkinson's disease (PD), Alzheimer's disease (AD), and Huntington's disease (HD). Table 1 shows reagents that modulate ferroptosis in animal models or patients of these CNS diseases. Thus ferroptosis inhibitors have exhibited great therapeutic potential for these CNS diseases. More and more efforts have been made to elucidate the role of ferroptosis in the pathogenesis of these diseases. We will elaborate on the relationship between ferroptosis and these CNS diseases below.

**TABLE 1:** Reagents that modulate ferroptosis in animal models or patients of CNS diseases.

CNS diseases	Reagents	Functions	References
Ischemic stroke	Extract of Naotaifang	Regulating TFR1/DMT1 and SCL7A11/GPX4 pathways	Lan et al. (2020)
	Carvacrol	Increasing the expression of GPX4	Guan et al. (2019)
	Tat-inked SelP Peptide	Upregulating the expression of GPX4	Alim et al. (2019)
Intracerebral hemorrhage stroke (ICH)	DFO	Chelating iron	Okauchi et al. (2010)
	Fer-1	Trapping free radicals	Li et al. (2017)
Parkinson's disease (PD)	FAC	Upgrading ferritin levels	Zhang et al. (2020)
	Fer-1	Trapping free radicals	Do Van et al. (2016)
	Deferiprone	Chelating iron	Martin-Bastida et al. (2017)
Alzheimer’s disease (AD)	Lip-1	Trapping free radicals	Hambright et al. (2017)
	Deferiprone	Chelating iron	Rao et al. (2020)
Huntington’s disease (HD)	DFO	Chelating iron	Chen et al. (2013)
	Fer-1 and its analogues	Trapping free radicals	Skouta et al. (2014)

### Stroke

In the United States, about 795,000 people experience a new or recurrent stroke each year (Benjamin et al., 2019). Of all strokes, 87% are ischemic stroke and 10% are intracerebral hemorrhage (ICH) stroke (Benjamin et al., 2019). Stroke usually leads to irreparable brain damage and the patients have to suffer from severe sequelae, such as hemiplegia, language impairment and cognitive impairment.

Ischemic stroke is caused by occlusion or contraction of blood vessels that restricts blood supply to certain parts of the brain (Barthels and Das, 2020). Insufficient blood in the brain fails to provide enough oxygen and nutrients to neurons, leading to their activation of the ischemic cascade, which is followed by excitotoxicity, oxidative stress, blood–brain barrier dysfunction, microvascular injury, hemostatic activation, post-ischemic inflammation and eventual cell death (Goossens and Hachimi-Idrissi, 2014). Before ferroptosis was identified, clinical studies had found that iron and oxidative stress could promote brain damage caused by ischemic stroke (Carbonell and Rama, 2007). Nowadays, increasing evidence reveals the relationship between ischemic stroke and ferroptosis. A recent study demonstrated that in acute ischemic stroke model of middle cerebral artery occlusion (MCAO) rats, neuronal ferroptosis was induced by the imbalance of iron metabolism and redox disorder (Lan et al., 2020). While the extract of Naotaifang, a compound Chinese herbal medicine, could suppress ferroptosis through TFR1/DMT1 and SCL7A11/GPX4 pathways, and then played a neuroprotective role on MCAO rats (Lan et al., 2020). This protective effect may be mediated by its active ingredients that can cross the blood-brain barrier and enter the brain tissue, but the blood brain permeability has not been examined. After cerebral ischemia, reperfusion is the most effective treatment. However, reperfusion will promote the production of ROS, increasing the damage and worsening the patients’ prognosis (Olmez and Ozyurt, 2012). Therefore, reducing brain ischemia/reperfusion injury is crucial in treating cerebral ischemia. Guan et al. (2019) found that the natural product carvacrol could inhibit ferroptosis by increasing the expression of GPX4, thereby exerting its protective effects on cognitive dysfunction in gerbils exposed to ischemia/reperfusion, but the blood brain permeability of carvacrol has not been examined. Additionally, Alim et al. (2019) created a Tat-linked SelP Peptide, which could greatly reduce the cerebral infarct volume caused by ischemia/reperfusion in mice. Mechanistically, this is because the Tat-linked SelP Peptide could block ferroptosis by driving transcriptional response to upregulate GPX4 in neurons.

Compared to ischemic stroke, ICH has a lower incidence (Benjamin et al., 2019), but it leads to higher mortality and more severe disability (An et al., 2017). ICH refers to bleeding into the brain due to rupture or leakage of blood vessels, leading to compression of brain tissue and neuronal damage. During this process, hemoglobin (Hb) and heme are released from the lysed erythrocytes. They are considered as neurotoxins because they can release iron and cause neuronal damage and death by enhancing the formation of ROS (Xiong et al., 2014). The iron in dead cells can also be absorbed by surrounding cells, causing even more catastrophic consequences (Xiong et al., 2014). The iron chelating agents DFO can effectively reduce ICH-induced neuronal damage in rats (Okauchi et al., 2010), and the cell death caused by ICH has the characteristics of ferroptosis *in vivo* and *in vitro* (Zille et al., 2017), all verifying that ferroptosis is closely related to ICH brain damage. Li et al. (2017) found that ferroptosis did occur in a mouse model of ICH and contributed to neuronal death. In addition, ferroptosis inhibitor Fer-1 can inhibit Hb-induced neuronal death in OHSCs.

In general, inhibiting ferroptosis can be a promising strategy for the prevention or treatments of stroke. However, no clinical trials that use ferroptosis inhibitors have been reported to treat stroke to date.

### PD

PD is the second most universal age-related neurodegenerative disease in the world (Elbaz et al., 2016). The clinical manifestations include resting tremor, muscle rigidity, gait and posture disorders (Cacabelos, 2017), which cause great pain and inconvenience to the patients and their families. Parkinson’s disease is characterized by the death of dopaminergic neurons, especially those in substantia nigra pars compacta (SNpc) and striatum (Cacabelos, 2017). The loss of dopaminergic neurons leads to insufficient secretion of dopamine, a pivotal neurotransmitter in the brain. Thus, the nerve conduction is blocked, leading to the symptoms of dyskinesia. Currently, dopamine-based therapies such as levodopa are used in clinic to relieve the motor symptoms in early PD (Katzenschlager and Lees, 2002). However, these treatments show severe side effects and have no improvement on the disease progression. Therefore, it is urgent to develop drugs that can slow or prevent the death of dopaminergic neurons in the brain.

The iron accumulation found on SNpc is one of the characteristics of PD patients, suggesting the link between iron and PD (Moreau et al., 2018). As a strong reducing agent, iron can not only cause ROS production in neurons, but also oxidize dopamine (Guiney et al., 2017). Increasing data have indicated that ferroptosis is an important pathway for the cell death of dopaminergic neurons and the occurrence of PD. Zhang et al. (2020) treated dopaminergic neurons MES23.5 cells with ferric ammonium citrate (FAC) to simulate the iron overload of PD, as FAC can upgrade ferritin levels in cells. They found ferroptosis occurred in the early stage of cell death, which was also detected in the PD mice. Furthermore, ferroptosis inhibitors also have a significant therapeutic effect on the PD mouse model. In 1-methyl-4-phenyl-1,2,3,6-tetrahydropyridine (MPTP)-treated mice, a well-established animal model of PD, Do Van et al. (2016) confirmed that the ferroptosis inhibitor Fer-1 could inhibit the death of dopaminergic neurons. Inspiringly, the results of a phase II clinical trial for PD patients (clinical trial NCT01539837) showed that treatment with iron chelator deferiprone (30 mg/kg) exhibited an improvement in motor symptoms and patients’ quality of life (Martin-Bastida et al., 2017).

### AD

AD is the most common type of irreversible dementia and a neurodegenerative disease that often occurs in the elderly. Its histological features are the accumulation of senile plaques composed of amyloid-β (Aβ) and neurofibrillary tangles (NFTs) formed by hyperphosphorylated tau protein in the memory and cognition area of the brain (Citron, 2010). AD is caused by the degradation of memory and cognition neurons, which may be the result of the interaction of genes and environment. The manifestations include behavioral changes, progressive memory loss, delusions, hallucinations and degradation in fine motor skills. Therefore, the patients are unable to live independently, bringing a heavy burden to the patients’ families and the society.

Before the definition of ferroptosis, abnormal iron metabolism and lipid peroxidation had been found to participate in the pathogenesis of AD (Obulesu et al., 2011). Evidence indicated that AD patients showed an excessive iron accumulation, which is more than 2 times the iron level observed in normal brains (Lovell et al., 1998). Accumulation of iron can not only prompt the accumulation and/or aggregation of the Aβ and tau protein, but also induces the ROS production in the brain of AD (Yamamoto et al., 2002). Oxidative stress is also reported to be an important pathological phenomenon that begins to appear early in the course of AD (Saito et al., 2019). When redox balance in the brain is impaired, oxidative stress can cause serious damage leading to AD. Moreover, oxidative stress has been reported to exacerbate AD pathology and cognitive dysfunction (Butterfield, 1997). Besides, it was indicated that 12/15-LOX was upregulated in the brain of AD patients, which may be related to the oxidative imbalance of AD (Pratico et al., 2004). Now increasing evidence implicates that ferroptosis may be involved in neuronal degeneration in AD. According to Morris water maze task, *Gpx4* ^KO^ mice showed obvious defects in spatial learning and memory function, while ferroptosis inhibitor Lip-1 could ameliorate the neurodegeneration in these mice (Hambright et al., 2017). Besides, a clinical measure on AD patients revealed that the level of GSH was reduced especially in the hippocampi (HP) and frontal cortices (FC), two vital brain regions related to the memory and cognition functions (Mandal et al., 2015).

Therapeutically, iron chelator desferrioxamine has already been conducted a clinical trial in AD in 1991 (Crapper McLachlan et al., 1991). A randomized, multi-center, double-blind Phase II trial using deferiprone for AD patients (clinical trial NCT03234686) is currently ongoing in Australia (Rao et al., 2020). Moreover, as mentioned above, Se can increase the resistance of cells to ferroptosis. It was reported that Se deficiency in the human body was associated with an increased risk of AD (Cardoso et al., 2014). However, in a phaseⅡclinical trial, though Se could be delivered into the CNS effectively by selenate, there were no significant effects on cognitive performance outcomes in AD patients. Therefore, the process of ferroptosis participating in AD needs further study, as AD may be the combination of many factors (Cardoso et al., 2019).

### HD

HD is an autosomal dominant neurodegenerative disease caused by the CAG repeat length mutation in the *huntington* gene (Ross and Tabrizi, 2011). It is characterized by highly selective and severe damage to the corpus striatum, resulting in dance-like movements, dystonia and progressive dementia. The mutant *huntington* may cause oxidative stress and neurotoxicity to the neurons in corpus striatum (Paul et al., 2014), which ultimately results in neuronal dysfunction and neuronal cell death, leading to patients with motor and cognitive impairments. However, the pathological mechanism of HD is complicated and has not been fully elucidated yet.

Some characteristics of ferroptosis have been observed in HD patients and experimental animal models, such as iron accumulation (Dominguez et al., 2016), lipid oxidation (Brocardo et al., 2016), oxidative stress (Pinho et al., 2020) and GSH redox cycle dysregulation (Ribeiro et al., 2012). For example, in R6/2 HD mouse brain, discrete puncta formed by iron accumulation was detected in the periplasmic cytoplasm of striated neurons by synchrotron X-ray fluorescence analysis (Chen et al., 2013). HD patients showed higher plasma lipid peroxidation level and lower GSH level (Klepac et al., 2007). Consistently, Kumar et al. (2010) found decreased GSH and GSH-S-transferase in the striatum, cortex and hippocampus in 3-nitropropionic acid-induced HD mouse. These phenomena imply that ferroptosis may play an important role in the pathogenesis of HD.

Stack et al. (2010) synthesized two triterpenoids derived from 2-cyano-3,12-dioxooleana-1,9-dien-28-oic acid (CDDO). They could reduce oxidative stress in the N171–82Q transgenic mouse model of HD, and improved their rotorod performance and survival. Mechanically, these two triterpenoids activated the NRF2/antioxidant response element (ARE) pathway and upregulated NRF2/ARE induced genes in the brain and peripheral tissues. Therefore, compounds targeting the NRF2/ARE pathway show great promise for the treatment of HD. Some ferroptosis regulators have also been found to work in HD models. For instance, intraventricular delivery of the iron chelator DFO led to an improvement in the motor phenotype of R6/2 HD mice (Chen et al., 2013). Skouta et al. (2014) found that the ferroptosis inhibitor Fer-1 and its analogues could prevent cell death in the brain slice model of HD.

## Discussion

Since ferroptosis was defined in 2012, the research on its mechanisms and clinical applications has been a hotspot. The currently known major regulatory mechanisms of ferroptosis involve intracellular lipid metabolism, iron metabolism, GSH-dependent pathway and CoQ_10_-dependent pathway as stated above. It is possible that certain novel regulators existing in ferroptosis have not been uncovered. And there are still many questions need to be solved regarding the mechanisms of ferroptosis and its relationship with diseases. For example, is there a final biomarker that executes the ferroptotic cell death and what is it? What other roles does mitochondrion play in ferroptosis except that TCA cycle promotes cystine deprivation-induced ferroptosis? Moreover, since much research on ferroptosis focuses on tumor cells, neuronal cells, kidney cells, and mouse embryonic fibroblasts, does ferroptosis occur in other cell types?

In addition to cancers, ferroptosis has also been reported to be associated with a variety of CNS diseases, and some ferroptosis inhibitors have achieved inspiring results in related animal models. Nonetheless, the role of ferroptosis in CNS diseases needs further elucidation, and currently there is no definitive evidence linking CNS diseases with ferroptosis in long-term animal model studies. Additionally, the existing small molecular regulators of ferroptosis are still limited, and have some disadvantages such as low stability and poor biocompatibility. Therefore, it is urgent to obtain potent ferroptosis inhibitors with good biocompatibility, strong stability and high safety by target-based or cell-based high-throughput screening, structural modification and other methods. The development of such compounds will be an important direction for the prevention and treatments of some human diseases such as CNS diseases.

In conclusion, the research on ferroptosis and its relationship with CNS diseases would be certainly potential for further understanding of the pathogenesis of these diseases and discovery of more effective therapeutic targets, although there are still many unsolved issues in the field. Since no effective therapeutic strategies toward CNS diseases, blockade of ferroptosis may be of value in the treatment of CNS diseases. Further insights into research related to ferroptosis are now likely to emerge rapidly.

## Glossary

ROS: reactive oxygen species
DFO: deferoxamine
PUFA,: polyunsaturated fatty acid
PL: phospholipid
*RPL8*: ribosomal protein L8
*IREB2*: iron response element binding protein 2
*ATP5G3*: ATP synthase F0 complex subunit C3
*CS*: citrate synthase
*TTC35*: tetratricopeptide repeat domain 35
*ACSF2*: acyl-CoA synthetase family member 2
*HSPB1*: heat shock protein β-1
*PTGS2*: prostaglandin-endoperoxide synthase 2
CNS: central nervous system
LOOH: lipid hydroperoxide
GSH: glutathione
CoQ_10_: coenzyme Q_10_
4-HNE: 4-hydroxy-2-nonenal
MDA: malondialdehyde
SFA: saturated fatty acid
MUFA: monounsaturated fatty acid
AA: arachidonic acid
AdA: adrenic acid
PE: phosphatidylethanolamine
ACSL4: acyl-CoA synthetase long-chain family member 4
LPCAT3: lysophosphatidylcholine acyltransferase 3
TZD: thiazolidinedione
TRO: troglitazone
PIO: pioglitazone
ROSI: rosiglitazone
LOX: lipoxygenase
L·: pentadienyl radical
·OH: hydroxyl radicals
LO·: alkoxy groups
HO_2_·: hydroperoxyl radicals
LOO·: peroxy radical
H_2_O_2_: hydrogen peroxide
RTA: radical trapping antioxidant
Fer-1: ferrostatin-1
Lip-1: liproxstatin-1
Tf: transferrin
TfR: transferrin receptor
DMT1: divalent metal transporter 1
LIP: labile iron pool
FT: ferritin
FTH1: ferritin heavy chain 1
FTL: ferritin light chain
NCOA4: nuclear receptor coactivator 4
FPN: ferroportin
GPX4: glutathione peroxidase 4
Se: selenium
L-OH: lipid alcohol
RSL3: Ras-selective lethal 3
Sec: selenocysteine
Glu: glutamate
Cys: cysteine
Gly: glycine
BSO: buthionine sulfoximine
GCL: glutamate-cysteine ligase
IPP: isopentenyl pyrophosphate
SQS: squalene synthase
HMG-CoA: 3-hydroxy-3-methyl glutaryl-coenzyme A
HMGCR: HMG-CoA reductase
FSP1: ferroptosis suppressor protein 1
NRF2: nuclear factor erythroid 2-related factor 2
TXNRD1: thioredoxin reductase 1
NADK: NAD^+^ kinase
NOX: NADPH oxidase
DPI: diphenylene iodonium
TCA cycle: tricarboxylic acid cycle
Keap1: Kelch-like ECH-associated protein
*NQO1*: NAD(P)H quinone oxidoreductase-1
*HO1*: heme oxygenase-1
ARF: auxin response factor
FOCAD: focadhesin
FAK: focal adhesion kinase
OHSC: organotypic hippocampal slice culture
PD: Parkinson’s disease
AD: Alzheimer’s disease
HD: Huntington’s disease
ICH: intracerebral hemorrhage
MCAO: middle cerebral artery occlusion
Hb: hemoglobin
SNpc: substantia nigra pars compacta
FAC: ferric ammonium citrate
MPTP: 1-methyl-4-phenyl-1,2,3,6-tetrahydropyridine
Aβ: amyloid-β
NFT: neurofibrillary tangle
HP: hippocampi
FC: frontal cortices
CDDO: 2-cyano-3,12-dioxooleana-1,9-dien-28-oic acid
ARE: antioxidant response element

## References

[B1] Agmon E. Solon J. Bassereau P. Stockwell B. R. (2018). Modeling the Effects of Lipid Peroxidation during Ferroptosis on Membrane Properties. Sci. Rep. 8, 5155. 10.1038/s41598-018-23408-0 29581451 PMC5979948

[B2] Alim I. Caulfield J. T. Chen Y. Swarup V. Geschwind D. H. Ivanova E. (2019). Selenium Drives a Transcriptional Adaptive Program to Block Ferroptosis and Treat Stroke. Cell 177, 1262–1279. 10.1016/j.cell.2019.03.032 31056284

[B3] An S. J. Kim T. J. Yoon B.-W. (2017). Epidemiology, Risk Factors, and Clinical Features of Intracerebral Hemorrhage: an Update. J. Stroke 19, 3–10. 10.5853/jos.2016.00864 28178408 PMC5307940

[B4] Angeli J. P. F. Shah R. Pratt D. A. Conrad M. (2017). Ferroptosis Inhibition: Mechanisms and Opportunities. Trends Pharmacol. Sci. 38, 489–498. 10.1016/j.tips.2017.02.005 28363764

[B5] Ayala A. Muñoz M. F. Argüelles S. (2014). Lipid Peroxidation: Production, Metabolism, and Signaling Mechanisms of Malondialdehyde and 4-Hydroxy-2-Nonenal. Oxidative Med. Cell Longevity 2014, 1–31. 10.1155/2014/360438 PMC4066722 24999379

[B6] Badgley M. A. Kremer D. M. Maurer H. C. DelGiorno K. E. Lee H.-J. Purohit V. (2020). Cysteine Depletion Induces Pancreatic Tumor Ferroptosis in Mice. Science 368, 85–89. 10.1126/science.aaw9872 32241947 PMC7681911

[B7] Bao W.-D. Pang P. Zhou X.-T. Hu F. Xiong W. Chen K. (2021). Loss of Ferroportin Induces Memory Impairment by Promoting Ferroptosis in Alzheimer's Disease. Cell Death Differ (in press). 10.1038/s41418-020-00685-9 PMC8166828 33398092

[B8] Barthels D. Das H. (2020). Current Advances in Ischemic Stroke Research and Therapies. Biochim. Biophys. Acta (Bba) - Mol. Basis Dis. 1866, 165260. 10.1016/j.bbadis.2018.09.012 PMC6981280 31699365

[B9] Benjamin E. J. Muntner P. Alonso A. Bittencourt M. S. Callaway C. W. Carson A. P. (2019). Heart Disease and Stroke Statistics-2019 Update: A Report from the American Heart Association. Circulation 139, e56. 10.1161/cir.0000000000000659 30700139

[B10] Bentinger M. Brismar K. Dallner G. (2007). The Antioxidant Role of Coenzyme Q. Mitochondrion 7, S41–S50. 10.1016/j.mito.2007.02.006 17482888

[B11] Bersuker K. Hendricks J. M. Li Z. Magtanong L. Ford B. Tang P. H. (2019). The CoQ Oxidoreductase FSP1 Acts Parallel to GPX4 to Inhibit Ferroptosis. Nature 575, 688–692. 10.1038/s41586-019-1705-2 31634900 PMC6883167

[B12] Bogdan A. R. Miyazawa M. Hashimoto K. Tsuji Y. (2016). Regulators of Iron Homeostasis: New Players in Metabolism, Cell Death, and Disease. Trends Biochem. Sci. 41, 274–286. 10.1016/j.tibs.2015.11.012 26725301 PMC4783254

[B13] Brash A. R. (1999). Lipoxygenases: Occurrence, Functions, Catalysis, and Acquisition of Substrate. J. Biol. Chem. 274, 23679–23682. 10.1074/jbc.274.34.23679 10446122

[B14] Brocardo P. S. McGinnis E. Christie B. R. Gil-Mohapel J. (2016). Time-course Analysis of Protein and Lipid Oxidation in the Brains of Yac128 Huntington's Disease Transgenic Mice. Rejuvenation Res. 19, 140–148. 10.1089/rej.2015.1736 26371883

[B15] Butterfield D. A. (1997). β-Amyloid-Associated Free Radical Oxidative Stress and Neurotoxicity: Implications for Alzheimer's Disease. Chem. Res. Toxicol. 10, 495–506. 10.1021/tx960130e 9168246

[B16] Cacabelos R. (2017). Parkinson's Disease: from Pathogenesis to Pharmacogenomics. Ijms 18, 551. 10.3390/ijms18030551 PMC5372567 28273839

[B17] Carbonell T. Rama R. (2007). Iron, Oxidative Stress and Early Neurological Deterioration in Ischemic Stroke. Cmc 14, 857–874. 10.2174/092986707780363014 17430141

[B18] Cardoso B. R. Bandeira V. S. Jacob-Filho W. Franciscato Cozzolino S. M. (2014). Selenium Status in Elderly: Relation to Cognitive Decline. J. Trace Elem. Med. Bio. 28, 422–426. 10.1016/j.jtemb.2014.08.009 25220532

[B19] Cardoso B. R. Hare D. J. Bush A. I. Roberts B. R. (2017). Glutathione Peroxidase 4: a New Player in Neurodegeneration?. Mol. Psychiatry 22, 328–335. 10.1038/mp.2016.196 27777421

[B20] Cardoso B. R. Roberts B. R. Malpas C. B. Vivash L. Genc S. Saling M. M. (2019). Supranutritional Sodium Selenate Supplementation Delivers Selenium to the central Nervous System: Results from a Randomized Controlled Pilot Trial in Alzheimer's Disease. Neurotherapeutics 16, 192–202. 10.1007/s13311-018-0662-z 30215171 PMC6361071

[B21] Chen D. Tavana O. Chu B. Erber L. Chen Y. Baer R. (2017). NRF2 Is a Major Target of ARF in P53-independent Tumor Suppression. Mol. Cel 68, 224–232 e4. 10.1016/j.molcel.2017.09.009 PMC5683418 28985506

[B22] Chen J. Marks E. Lai B. Zhang Z. Duce J. A. Lam L. Q. (2013). Iron Accumulates in Huntington's Disease Neurons: protection by Deferoxamine. Plos One 8, e77023. 10.1371/journal.pone.0077023 24146952 PMC3795666

[B23] Citron M. (2010). Alzheimer's Disease: Strategies for Disease Modification. Nat. Rev. Drug Discov. 9, 387–398. 10.1038/nrd2896 20431570

[B24] Derry P. J. Hegde M. L. Jackson G. R. Kayed R. Tour J. M. Tsai A.-L. (2020). Revisiting the Intersection of Amyloid, Pathologically Modified Tau and Iron in Alzheimer's Disease from a Ferroptosis Perspective. Prog. Neurobiol. 184, 101716. 10.1016/j.pneurobio.2019.101716 31604111 PMC7850812

[B25] Dixon S. J. Lemberg K. M. Lamprecht M. R. Skouta R. Zaitsev E. M. Gleason C. E. (2012). Ferroptosis: an Iron-dependent Form of Nonapoptotic Cell Death. Cell 149, 1060–1072. 10.1016/j.cell.2012.03.042 22632970 PMC3367386

[B26] Do Van B. Gouel F. Jonneaux A. Timmerman K. Gelé P. Pétrault M. (2016). Ferroptosis, a Newly Characterized Form of Cell Death in Parkinson's Disease that Is Regulated by PKC. Neurobiol. Dis. 94, 169–178. 10.1016/j.nbd.2016.05.011 27189756

[B27] Doll S. Freitas F. P. Shah R. Aldrovandi M. da Silva M. C. Ingold I. (2019). FSP1 Is a Glutathione-independent Ferroptosis Suppressor. Nature 575, 693–698. 10.1038/s41586-019-1707-0 31634899

[B28] Doll S. Proneth B. Tyurina Y. Y. Panzilius E. Kobayashi S. Ingold I. (2017). ACSL4 Dictates Ferroptosis Sensitivity by Shaping Cellular Lipid Composition. Nat. Chem. Biol. 13, 91–98. 10.1038/nchembio.2239 27842070 PMC5610546

[B29] Domínguez J. F. Ng A. C. Poudel G. Stout J. C. Churchyard A. Chua P. (2016). Iron Accumulation in the Basal Ganglia in Huntington's Disease: Cross-Sectional Data from the IMAGE-HD Study. J. Neurol. Neurosurg. Psychiatry 87, 545–549. 10.1136/jnnp-2014-310183 25952334

[B30] Elbaz A. Carcaillon L. Kab S. Moisan F. (2016). Epidemiology of Parkinson's Disease. Revue Neurologique 172, 14–26. 10.1016/j.neurol.2015.09.012 26718594

[B31] Else P. L. (2017). Membrane Peroxidation in Vertebrates: Potential Role in Metabolism and Growth. Eur. J. Lipid Sci. Technol. 119, 1600319. 10.1002/ejlt.201600319

[B32] Fink S. L. Cookson B. T. (2005). Apoptosis, Pyroptosis, and Necrosis: Mechanistic Description of Dead and Dying Eukaryotic Cells. Iai 73, 1907–1916. 10.1128/iai.73.4.1907-1916.2005 PMC1087413 15784530

[B33] Friedmann Angeli J. P. Schneider M. Proneth B. Tyurina Y. Y. Tyurin V. A. Hammond V. J. (2014). Inactivation of the Ferroptosis Regulator Gpx4 Triggers Acute Renal Failure in Mice. Nat. Cel Biol. 16, 1180–1191. 10.1038/ncb3064 PMC4894846 25402683

[B34] Friedmann Angeli J. P. Conrad M. (2018). Selenium and GPX4, a Vital Symbiosis. Free Radic. Biol. Med. 127, 153–159. 10.1016/j.freeradbiomed.2018.03.001 29522794

[B35] Gao M. Monian P. Pan Q. Zhang W. Xiang J. Jiang X. (2016). Ferroptosis Is an Autophagic Cell Death Process. Cell Res 26, 1021–1032. 10.1038/cr.2016.95 27514700 PMC5034113

[B36] Gao M. Monian P. Quadri N. Ramasamy R. Jiang X. (2015). Glutaminolysis and Transferrin Regulate Ferroptosis. Mol. Cel 59, 298–308. 10.1016/j.molcel.2015.06.011 PMC4506736 26166707

[B37] Gao M. Yi J. Zhu J. Minikes A. M. Monian P. Thompson C. B. (2019). Role of Mitochondria in Ferroptosis. Mol. Cel 73, 354–363. 10.1016/j.molcel.2018.10.042 PMC6338496 30581146

[B38] Gaschler M. M. Stockwell B. R. (2017). Lipid Peroxidation in Cell Death. Biochem. Biophysical Res. Commun. 482, 419–425. 10.1016/j.bbrc.2016.10.086 PMC5319403 28212725

[B39] Geng N. Shi B. J. Li S. L. Zhong Z. Y. Li Y. C. Xua W. L. (2018). Knockdown of Ferroportin Accelerates Erastin-Induced Ferroptosis in Neuroblastoma Cells. Eur. Rev. Med. Pharmacol. Sci. 22, 3826–3836. 10.26355/eurrev_201806_15267 29949159

[B40] Goossens J. Hachimi-Idrissi S. (2014). Combination of Therapeutic Hypothermia and Other Neuroprotective Strategies after an Ischemic Cerebral Insult. Cn 12, 399–412. 10.2174/1570159x12666140424233036 PMC4243031 25426009

[B41] Guan X. Li X. Yang X. Yan J. Shi P. Ba L. (2019). The Neuroprotective Effects of Carvacrol on Ischemia/reperfusion-Induced Hippocampal Neuronal Impairment by Ferroptosis Mitigation. Life Sci. 235, 116795. 10.1016/j.lfs.2019.116795 31470002

[B42] Guiney S. J. Adlard P. A. Bush A. I. Finkelstein D. I. Ayton S. (2017). Ferroptosis and Cell Death Mechanisms in Parkinson's Disease. Neurochem. Int. 104, 34–48. 10.1016/j.neuint.2017.01.004 28082232

[B43] Hambright W. S. Fonseca R. S. Chen L. Na R. Ran Q. (2017). Ablation of Ferroptosis Regulator Glutathione Peroxidase 4 in Forebrain Neurons Promotes Cognitive Impairment and Neurodegeneration. Redox Biol. 12, 8–17. 10.1016/j.redox.2017.01.021 28212525 PMC5312549

[B44] Harrison P. M. Arosio P. (1996). The Ferritins: Molecular Properties, Iron Storage Function and Cellular Regulation. Biochim. Biophys. Acta (Bba) - Bioenerg. 1275, 161–203. 10.1016/0005-2728(96)00022-9 8695634

[B45] Hassannia B. Vandenabeele P. Vanden Berghe T. (2019). Targeting Ferroptosis to Iron Out Cancer. Cancer Cell 35, 830–849. 10.1016/j.ccell.2019.04.002 31105042

[B46] Hassannia B. Wiernicki B. Ingold I. Qu F. Van Herck S. Tyurina Y. Y. (2018). Nano-targeted Induction of Dual Ferroptotic Mechanisms Eradicates High-Risk Neuroblastoma. J. Clin. Invest. 128, 3341–3355. 10.1172/jci99032 29939160 PMC6063467

[B47] Hayano M. Yang W. S. Corn C. K. Pagano N. C. Stockwell B. R. (2016). Loss of Cysteinyl-tRNA Synthetase (CARS) Induces the Transsulfuration Pathway and Inhibits Ferroptosis Induced by Cystine Deprivation. Cel Death Differ 23, 270–278. 10.1038/cdd.2015.93 PMC4716307 26184909

[B48] Hirschhorn T. Stockwell B. R. (2019). The Development of the Concept of Ferroptosis. Free Radic. Biol. Med. 133, 130–143. 10.1016/j.freeradbiomed.2018.09.043 30268886 PMC6368883

[B49] Ingold I. Berndt C. Schmitt S. Doll S. Poschmann G. Buday K. (2018). Selenium Utilization by GPX4 Is Required to Prevent Hydroperoxide-Induced Ferroptosis. Cell 172, 409–422.e21. 10.1016/j.cell.2017.11.048 29290465

[B50] Islam Q. T. Sayers D. E. Theil E. C. (1989). Studies of Temperature Dependence of Iron Environment in an Undecairon(III) Oxo-Hydroxo Aggregate Compound Compared to Horse Spleen Ferritin. Physica B: Condensed Matter 158, 99–100. 10.1016/0921-4526(89)90213-5

[B51] Ji C. Kosman D. J. (2015). Molecular Mechanisms of Non-transferrin-bound and Transferring-Bound Iron Uptake in Primary Hippocampal Neurons. J. Neurochem. 133, 668–683. 10.1111/jnc.13040 25649872 PMC4406823

[B52] Jiang L. Kon N. Li T. Wang S.-J. Su T. Hibshoosh H. (2015). Ferroptosis as a P53-Mediated Activity during Tumour Suppression. Nature 520, 57–62. 10.1038/nature14344 25799988 PMC4455927

[B53] Kagan V. E. Mao G. Qu F. Angeli J. P. F. Doll S. Croix C. S. (2017). Oxidized Arachidonic and Adrenic PEs Navigate Cells to Ferroptosis. Nat. Chem. Biol. 13, 81–90. 10.1038/nchembio.2238 27842066 PMC5506843

[B54] Kajarabille N. Latunde-Dada G. O. (2019). Programmed Cell-Death by Ferroptosis: Antioxidants as Mitigators. Ijms 20, 4968. 10.3390/ijms20194968 PMC6801403 31597407

[B55] Kamata T. (2009). Roles of Nox1 and Other Nox Isoforms in Cancer Development. Cancer Sci. 100, 1382–1388. 10.1111/j.1349-7006.2009.01207.x 19493276 PMC11158854

[B56] Katzenschlager R. Lees A. J. (2002). Treatment of Parkinson's Disease: Levodopa as the First Choice. J. Neurol. 249 (Suppl. 2), II19-24. 10.1007/s00415-002-1204-4 12375059

[B57] Klepac N. Relja M. Klepac R. Hećimović S. Babić T. Trkulja V. (2007). Oxidative Stress Parameters in Plasma of Huntington's Disease Patients, Asymptomatic Huntington's Disease Gene Carriers and Healthy Subjects. J. Neurol. 254, 1676–1683. 10.1007/s00415-007-0611-y 17990062

[B58] Kuhn H. Banthiya S. van Leyen K. (2015). Mammalian Lipoxygenases and Their Biological Relevance. Biochim. Biophys. Acta (Bba) - Mol. Cel Biol. Lipids 1851, 308–330. 10.1016/j.bbalip.2014.10.002 PMC4370320 25316652

[B59] Kumar P. Kalonia H. Kumar A. (2010). Nitric Oxide Mechanism in the Protective Effect of Antidepressants against 3-nitropropionic Acid-Induced Cognitive Deficit, Glutathione and Mitochondrial Alterations in Animal Model of Huntington's Disease. Behav. Pharmacol. 21, 217–230. 10.1097/fbp.0b013e32833a5bf4 20480544

[B60] Lan B. Ge J.-W. Cheng S.-W. Zheng X.-L. Liao J. He C. (2020). Extract of Naotaifang, a Compound Chinese Herbal Medicine, Protects Neuron Ferroptosis Induced by Acute Cerebral Ischemia in Rats. J. Integr. Med. 18, 344–350. 10.1016/j.joim.2020.01.008 32107172

[B61] Li Q. Han X. Lan X. Gao Y. Wan J. Durham F. (2017). Inhibition of Neuronal Ferroptosis Protects Hemorrhagic Brain. JCI Insight 2, e90777. 10.1172/jci.insight.90777 28405617 PMC5374066

[B62] Li Q. Li Q.-Q. Jia J.-N. Sun Q.-Y. Zhou H.-H. Jin W.-L. (2019). Baicalein Exerts Neuroprotective Effects in FeCl3-Induced Posttraumatic Epileptic Seizures via Suppressing Ferroptosis. Front. Pharmacol. 10, 38. 10.3389/fphar.2019.00638 31231224 PMC6568039

[B63] Li Y. Zeng X. Lu D. Yin M. Shan M. Gao Y. (2021). Erastin Induces Ferroptosis via Ferroportin-Mediated Iron Accumulation in Endometriosis. Hum. Reprod. 36, 951–964. 10.1093/humrep/deaa363 33378529

[B64] Liu P. Wu D. Duan J. Xiao H. Zhou Y. Zhao L. (2020). NRF2 Regulates the Sensitivity of Human NSCLC Cells to Cystine Deprivation-Induced Ferroptosis via FOCAD-FAK Signaling Pathway. Redox Biol. 37, 101702. 10.1016/j.redox.2020.101702 32898818 PMC7486457

[B65] Llabani E. Hicklin R. W. Lee H. Y. Motika S. E. Crawford L. A. Weerapana E. (2019). Diverse Compounds from Pleuromutilin lead to a Thioredoxin Inhibitor and Inducer of Ferroptosis. Nat. Chem. 11, 521–532. 10.1038/s41557-019-0261-6 31086302 PMC6639018

[B66] Lovell M. A. Robertson J. D. Teesdale W. J. Campbell J. L. Markesbery W. R. (1998). Copper, Iron and Zinc in Alzheimer's Disease Senile Plaques. J. Neurol. Sci. 158, 47–52. 10.1016/s0022-510x(98)00092-6 9667777

[B67] Ma D. Li C. Jiang P. Jiang Y. Wang J. Zhang D. (2020). Inhibition of Ferroptosis Attenuates Acute Kidney Injury in Rats with Severe Acute Pancreatitis. Dig. Dis. Sci. 66, 483–492. 10.1007/s10620-020-06225-2 32219613

[B68] Magtanong L. Dixon S. J. (2018). Ferroptosis and Brain Injury. Dev. Neurosci. 40, 382–395. 10.1159/000496922 30820017 PMC6658337

[B69] Mandal P. K. Saharan S. Tripathi M. Murari G. (2015). Brain Glutathione Levels - a Novel Biomarker for Mild Cognitive Impairment and Alzheimer's Disease. Biol. Psychiatry 78, 702–710. 10.1016/j.biopsych.2015.04.005 26003861

[B70] Martin-Bastida A. Ward R. J. Newbould R. Piccini P. Sharp D. Kabba C. (2017). Brain Iron Chelation by Deferiprone in a Phase 2 Randomised Double-Blinded Placebo Controlled Clinical Trial in Parkinson's Disease. Sci. Rep. 7, 1398. 10.1038/s41598-017-01402-2 28469157 PMC5431100

[B71] Mclachlan D. Dalton A. J. Kruck T. P. Bell M. Y. Smith W. L. Kalow W. (1991). Intramuscular Desferrioxamine in Patients with Alzheimer's Disease. The Lancet 337, 1304–1308. 10.1016/0140-6736(91)92978-b 1674295

[B72] Moosmann B. Behl C. (2004). Selenoproteins, Cholesterol-Lowering Drugs, and the Consequences Revisiting of the Mevalonate Pathway. Trends Cardiovasc. Med. 14, 273–281. 10.1016/j.tcm.2004.08.003 15542379

[B73] Moreau C. Duce J. A. Rascol O. Devedjian J.-C. Berg D. Dexter D. (2018). Iron as a Therapeutic Target for Parkinson's Disease. Mov Disord. 33, 568–574. 10.1002/mds.27275 29380903

[B74] Mou Y. Wang J. Wu J. He D. Zhang C. Duan C. (2019). Ferroptosis, a New Form of Cell Death: Opportunities and Challenges in Cancer. J. Hematol. Oncol. 12, 34. 10.1186/s13045-019-0720-y 30925886 PMC6441206

[B75] Nordberg J. Arnér E. S. J. (2001). Reactive Oxygen Species, Antioxidants, and the Mammalian Thioredoxin System1 1This Review Is Based on the Licentiate Thesis "Thioredoxin Reductase-Interactions with the Redox Active Compounds 1-Chloro-2,4-Dinitrobenzene and Lipoic Acid" by Jonas Nordberg, 2001, Karolinska Institute, Stockholm, ISBN 91-631-1064-4. Free Radic. Biol. Med. 31 (11), 1287–1312. 10.1016/s0891-5849(01)00724-9 11728801

[B76] Obulesu M. Venu R. Somashekhar R. (2011). Lipid Peroxidation in Alzheimer's Disease: Emphasis on Metal-Mediated Neurotoxicity. Acta Neurol. Scand. 124, 295–301. 10.1111/j.1600-0404.2010.01483.x 21303349

[B77] Okauchi M. Hua Y. Keep R. F. Morgenstern L. B. Schallert T. Xi G. (2010). Deferoxamine Treatment for Intracerebral Hemorrhage in Aged Rats. Stroke 41, 375–382. 10.1161/strokeaha.109.569830 20044521 PMC2896218

[B78] Olmez I. Ozyurt H. (2012). Reactive Oxygen Species and Ischemic Cerebrovascular Disease. Neurochem. Int. 60, 208–212. 10.1016/j.neuint.2011.11.009 22122807

[B79] Paul B. D. Sbodio J. I. Xu R. Vandiver M. S. Cha J. Y. Snowman A. M. (2014). Cystathionine γ-lyase Deficiency Mediates Neurodegeneration in Huntington's Disease. Nature 509, 96–100. 10.1038/nature13136 24670645 PMC4349202

[B80] Pinho B. R. Duarte A. I. Canas P. M. Moreira P. I. Murphy M. P. Oliveira J. M. A. (2020). The Interplay between Redox Signalling and Proteostasis in Neurodegeneration: *In Vivo* Effects of a Mitochondria-Targeted Antioxidant in Huntington's Disease Mice. Free Radic. Biol. Med. 146, 372–382. 10.1016/j.freeradbiomed.2019.11.021 31751762 PMC6970224

[B81] Praticò D. Zhukareva V. Yao Y. Uryu K. Funk C. D. Lawson J. A. (2004). 12/15-Lipoxygenase Is Increased in Alzheimer's Disease. Am. J. Pathol. 164, 1655–1662. 10.1016/s0002-9440(10)63724-8 15111312 PMC1615676

[B82] Rao S. S. Portbury S. D. Lago L. Bush A. I. Adlard P. A. (2020). The Iron Chelator Deferiprone Improves the Phenotype in a Mouse Model of Tauopathy1. Jad 77, 753–771. 10.3233/jad-200551 32741833

[B83] Ribeiro M. Rosenstock T. R. Cunha-Oliveira T. Ferreira I. L. Oliveira C. R. Rego A. C. (2012). Glutathione Redox Cycle Dysregulation in Huntington's Disease Knock-In Striatal Cells. Free Radic. Biol. Med. 53, 1857–1867. 10.1016/j.freeradbiomed.2012.09.004 22982598

[B84] Ross C. A. Tabrizi S. J. (2011). Huntington's Disease: from Molecular Pathogenesis to Clinical Treatment. Lancet Neurol. 10, 83–98. 10.1016/s1474-4422(10)70245-3 21163446

[B85] Saito T. Hisahara S. Iwahara N. Emoto M. C. Yokokawa K. Suzuki H. (2019). Early Administration of Galantamine from Preplaque Phase Suppresses Oxidative Stress and Improves Cognitive Behavior in APPswe/PS1dE9 Mouse Model of Alzheimer's Disease. Free Radic. Biol. Med. 145, 20–32. 10.1016/j.freeradbiomed.2019.09.014 31536772

[B86] Sato H. Tamba M. Ishii T. Bannai S. (1999). Cloning and Expression of a Plasma Membrane Cystine/glutamate Exchange Transporter Composed of Two Distinct Proteins. J. Biol. Chem. 274, 11455–11458. 10.1074/jbc.274.17.11455 10206947

[B87] Shimada K. Hayano M. Pagano N. C. Stockwell B. R. (2016a). Cell-line Selectivity Improves the Predictive Power of Pharmacogenomic Analyses and Helps Identify NADPH as Biomarker for Ferroptosis Sensitivity. Cel Chem. Biol. 23, 225–235. 10.1016/j.chembiol.2015.11.016 PMC4792701 26853626

[B88] Shimada K. Skouta R. Kaplan A. Yang W. S. Hayano M. Dixon S. J. (2016b). Global Survey of Cell Death Mechanisms Reveals Metabolic Regulation of Ferroptosis. Nat. Chem. Biol. 12, 497–503. 10.1038/nchembio.2079 27159577 PMC4920070

[B89] Shin D. Lee J. You J. H. Kim D. Roh J.-L. (2020). Dihydrolipoamide Dehydrogenase Regulates Cystine Deprivation-Induced Ferroptosis in Head and Neck Cancer. Redox Biol. 30, 101418. 10.1016/j.redox.2019.101418 31931284 PMC6957841

[B90] Skouta R. Dixon S. J. Wang J. Dunn D. E. Orman M. Shimada K. (2014). Ferrostatins Inhibit Oxidative Lipid Damage and Cell Death in Diverse Disease Models. J. Am. Chem. Soc. 136, 4551–4556. 10.1021/ja411006a 24592866 PMC3985476

[B91] Stack C. Ho D. Wille E. Calingasan N. Y. Williams C. Liby K. (2010). Triterpenoids CDDO-Ethyl Amide and CDDO-Trifluoroethyl Amide Improve the Behavioral Phenotype and Brain Pathology in a Transgenic Mouse Model of Huntington's Disease. Free Radic. Biol. Med. 49, 147–158. 10.1016/j.freeradbiomed.2010.03.017 20338236 PMC2916021

[B92] Sun X. Ou Z. Chen R. Niu X. Chen D. Kang R. (2016). Activation of the P62-Keap1-NRF2 Pathway Protects against Ferroptosis in Hepatocellular Carcinoma Cells. Hepatology 63, 173–184. 10.1002/hep.28251 26403645 PMC4688087

[B93] Sun X. Ou Z. Xie M. Kang R. Fan Y. Niu X. (2015). HSPB1 as a Novel Regulator of Ferroptotic Cancer Cell Death. Oncogene 34, 5617–5625. 10.1038/onc.2015.32 25728673 PMC4640181

[B94] Sun Y. Zheng Y. Wang C. Liu Y. (2018). Glutathione Depletion Induces Ferroptosis, Autophagy, and Premature Cell Senescence in Retinal Pigment Epithelial Cells. Cell Death Dis 9, 753. 10.1038/s41419-018-0794-4 29988039 PMC6037763

[B95] Wang L. Cai H. Hu Y. Liu F. Huang S. Zhou Y. (2018). A Pharmacological Probe Identifies Cystathionine β-synthase as a New Negative Regulator for Ferroptosis. Cel Death Dis 9, 1005. 10.1038/s41419-018-1063-2 PMC6158189 30258181

[B96] Warner G. J. Berry M. J. Moustafa M. E. Carlson B. A. Hatfield D. L. Faust J. R. (2000). Inhibition of Selenoprotein Synthesis by Selenocysteine tRNA[Ser]Sec Lacking Isopentenyladenosine. J. Biol. Chem. 275, 28110–28119. 10.1074/jbc.M001280200 10821829

[B97] Weiland A. Wang Y. Wu W. Lan X. Han X. Li Q. (2019). Ferroptosis and its Role in Diverse Brain Diseases. Mol. Neurobiol. 56, 4880–4893. 10.1007/s12035-018-1403-3 30406908 PMC6506411

[B98] Wolpaw A. J. Shimada K. Skouta R. Welsch M. E. Akavia U. D. Pe'er D. (2011). Modulatory Profiling Identifies Mechanisms of Small Molecule-Induced Cell Death. Proc. Natl. Acad. Sci. 108, E771–E780. 10.1073/pnas.1106149108 21896738 PMC3182736

[B99] Xie Y. Hou W. Song X. Yu Y. Huang J. Sun X. (2016). Ferroptosis: Process and Function. Cel Death Differ 23, 369–379. 10.1038/cdd.2015.158 PMC5072448 26794443

[B100] Xiong X.-Y. Wang J. Qian Z.-M. Yang Q.-W. (2014). Iron and Intracerebral Hemorrhage: from Mechanism to Translation. Transl. Stroke Res. 5, 429–441. 10.1007/s12975-013-0317-7 24362931

[B101] Yagoda N. von Rechenberg M. Zaganjor E. Bauer A. J. Yang W. S. Fridman D. J. (2007). Ras-raf-mek-dependent Oxidative Cell Death Involving Voltage-dependent Anion Channels. Nature 447, 865–869. 10.1038/nature05859 PMC3047570 17568748

[B102] Yamamoto A. Shin R.-W. Hasegawa K. Naiki H. Sato H. Yoshimasu F. (2002). Iron (III) Induces Aggregation of Hyperphosphorylated τ and its Reduction to Iron (II) Reverses the Aggregation: Implications in the Formation of Neurofibrillary Tangles of Alzheimer's Disease. J. Neurochem. 82, 1137–1147. 10.1046/j.1471-4159.2002.t01-1-01061.x 12358761

[B103] Yang W. S. SriRamaratnam R. Welsch M. E. Shimada K. Skouta R. Viswanathan V. S. (2014). Regulation of Ferroptotic Cancer Cell Death by GPX4. Cell 156, 317–331. 10.1016/j.cell.2013.12.010 24439385 PMC4076414

[B104] Yang W. S. Stockwell B. R. (2016). Ferroptosis: Death by Lipid Peroxidation. Trends Cel Biol. 26, 165–176. 10.1016/j.tcb.2015.10.014 PMC4764384 26653790

[B105] Yang W. S. Stockwell B. R. (2008). Synthetic Lethal Screening Identifies Compounds Activating Iron-dependent, Nonapoptotic Cell Death in Oncogenic-RAS-Harboring Cancer Cells. Chem. Biol. 15, 234–245. 10.1016/j.chembiol.2008.02.010 18355723 PMC2683762

[B106] Yin H. Xu L. Porter N. A. (2011). Free Radical Lipid Peroxidation: Mechanisms and Analysis. Chem. Rev. 111, 5944–5972. 10.1021/cr200084z 21861450

[B107] Zhang P. Chen L. Zhao Q. Du X. Bi M. Li Y. (2020). Ferroptosis Was More Initial in Cell Death Caused by Iron Overload and its Underlying Mechanism in Parkinson's Disease. Free Radic. Biol. Med. 152, 227–234. 10.1016/j.freeradbiomed.2020.03.015 32217194

[B108] Zhong H. Yin H. (2015). Role of Lipid Peroxidation Derived 4-hydroxynonenal (4-HNE) in Cancer: Focusing on Mitochondria. Redox Biol. 4, 193–199. 10.1016/j.redox.2014.12.011 25598486 PMC4803793

[B109] Zille M. Karuppagounder S. S. Chen Y. Gough P. J. Bertin J. Finger J. (2017). Neuronal Death after Hemorrhagic Stroke *In Vitro* and *In Vivo* Shares Features of Ferroptosis and Necroptosis. Stroke 48, 1033–1043. 10.1161/strokeaha.116.015609 28250197 PMC5613764

[B110] Zou Y. Palte M. J. Deik A. A. Li H. Eaton J. K. Wang W. (2019). A GPX4-dependent Cancer Cell State Underlies the clear-cell Morphology and Confers Sensitivity to Ferroptosis. Nat. Commun. 10, 1617. 10.1038/s41467-019-09277-9 30962421 PMC6453886

